# Covalent Organic Frameworks with Ionic Liquid-Moieties (ILCOFs): Structures, Synthesis, and CO_2_ Conversion

**DOI:** 10.3390/nano12203615

**Published:** 2022-10-15

**Authors:** Ruina Zhang, Zekai Zhang, Quanli Ke, Bing Zhou, Guokai Cui, Hanfeng Lu

**Affiliations:** College of Chemical Engineering, Zhejiang University of Technology, Huzhou 313299, China

**Keywords:** porous materials, ionic COF, polymer, reduction, cycloaddition, CCUS, greenhouse gas control, CO_2_ capture, CO_2_-philic sorbent, carbon neutral

## Abstract

CO_2_, an acidic gas, is usually emitted from the combustion of fossil fuels and leads to the formation of acid rain and greenhouse effects. CO_2_ can be used to produce kinds of value-added chemicals from a viewpoint based on carbon capture, utilization, and storage (CCUS). With the combination of unique structures and properties of ionic liquids (ILs) and covalent organic frameworks (COFs), covalent organic frameworks with ionic liquid-moieties (ILCOFs) have been developed as a kind of novel and efficient sorbent, catalyst, and electrolyte since 2016. In this critical review, we first focus on the structures and synthesis of different kinds of ILCOFs materials, including ILCOFs with IL moieties located on the main linkers, on the nodes, and on the side chains. We then discuss the ILCOFs for CO_2_ capture and conversion, including the reduction and cycloaddition of CO_2_. Finally, future directions and prospects for ILCOFs are outlined. This review is beneficial for academic researchers in obtaining an overall understanding of ILCOFs and their application of CO_2_ conversion. This work will open a door to develop novel ILCOFs materials for the capture, separation, and utilization of other typical acid, basic, or neutral gases such as SO_2_, H_2_S, NOx, NH_3_, and so on.

## 1. Introduction

Emitted from the combustion of fossil fuels in power plants, a large amount of CO_2_ in the atmosphere leads to the greenhouse effect and global warming. It is reported by the World Meteorological Organization (WMO) in the “State of the Global Climate 2021” that the global mean temperature in 2021 was around 1.11 ± 0.13 °C above the 1850–1900 pre-industrial average, and the concentration of CO_2_ reached 413.2 ± 0.2 ppm (2020) [1]. This results in harm to human life and the social economy. Carbon capture, utilization, and storage (CCUS) is a way of reducing carbon emissions involving CO_2_ capture from high-emission sources and the air, CO_2_ transportation from sources to sinks, and the reuse or permanent storage of the captured CO_2_ [2]. Kinds of CCUS technologies continue to be developed. Due to its high reactivity with CO_2_, monoethanolamine (MEA) has been used in industrial processes to chemically capture CO_2_ for many years [3]. However, this method also has the problems of volatility and serious equipment corrosion.

Ionic Liquids (ILs) are a kind of organic compound; they are composed of organic cations and organic or inorganic anions [4,5]. Typical cations include imidazolium, pyridinium, quaternary ammonium, quaternary phosphonium, etc., while typical anions include acidic anions such as halogen anions ([X]^–^), tetrafluoroborate ([BF_4_]^–^), hexafluorophosphate ([PF_6_]^–^), and bis(trifluoromethanesulfonyl)imide ([TFSI]^–^) and basic anions such as aprotic heterocyclic anions ([AHA]^–^) and phenolate anions. It is known that ILs are always liquid at room temperature or below 100 °C [6]. ILs have received much more attention because of their unique properties such as low vapor pressure, high chemical stability, wide liquid temperature range, and tunable structure-properties; they are applied as solvents and catalysts in many fields, such as the energy and environment [7,8,9], chemistry and materials synthesis [10,11,12,13], and pharmaceutics and medicine fields [14,15,16,17]. In addition, various kinds of porous organic materials have been developed, including metal-organic frameworks (MOFs) [18], covalent organic frameworks (COFs) [19,20], covalent triazine frameworks (CTFs) [21], and other amorphous porous organic polymers (POPs) [22]. These porous materials can also be applied as sorbents or catalysts in the fields of energy [23,24,25], environment [26,27,28,29], chemistry and materials synthesis [30,31,32], and pharmaceutics and medicine [33,34,35]. Among these porous materials, COFs have received increasing attention as a class of crystalline porous organic polymers with permanent porosity and highly ordered structures, containing covalently connected molecular building units [36,37]. COFs have also received increasing attention as sorbents, supports, and catalysts [38,39]. Based on the unique properties of ILs and COFs, covalent organic frameworks with ionic liquid-moieties (ILCOFs) were developed as novel porous ionic materials used in many fields [40,41]. The methods of the design and synthesis of ILCOFs are based on the synthesis strategies of ILs and COFs, including direct methods (pre-synthesis) and indirect methods (post-synthesis). Furthermore, it is known that the functionalized ILs containing active sites on cations or anions are efficient for CCUS [42,43,44]. Thus, ILCOFs would obtain a high efficiency on CCUS.

There are many review articles that have been published for ILs, COFs, or ionic COFs, and a few of them focused on CCUS. However, none of the review articles discussed the ILCOFs from a viewpoint of ILs. Some recent examples include: Zhang et al. [45] reviewed the IL-based CO_2_ capture systems from the structure and interaction to the process. Zhou and Wang et al. [46] focused on the synthesis of porous poly(ionic liquid)s for chemical CO_2_ fixation with epoxides. Jiang et al. [36] reviewed the COFs from the design and synthesis to the functions. Islam et al. [47] reported a study of contemporary progress relating to COF materials for CO_2_ capture and fixation reactions. Recently, Zhang et al. [48] focused on the design and application of ionic COFs. However, with the development of ILCOFs and the increasing attention on ILs, it is crucial to review this field from a viewpoint of ILs.

In this critical review, we mainly focus our attention on the recent advances of (1) the structures and synthesis of different kinds of ILCOFs materials, including ILCOFs with IL moieties located on the main linkers, on the nodes, and on the side chains, from a viewpoint of ILs, and (2) the ILCOFs for CO_2_ conversion, including the reduction and cycloaddition of CO_2_. Finally, future directions and prospects for ILCOFs are outlined.

## 2. Classification, Structures, and Synthesis of ILCOFs

**Figure 1:**
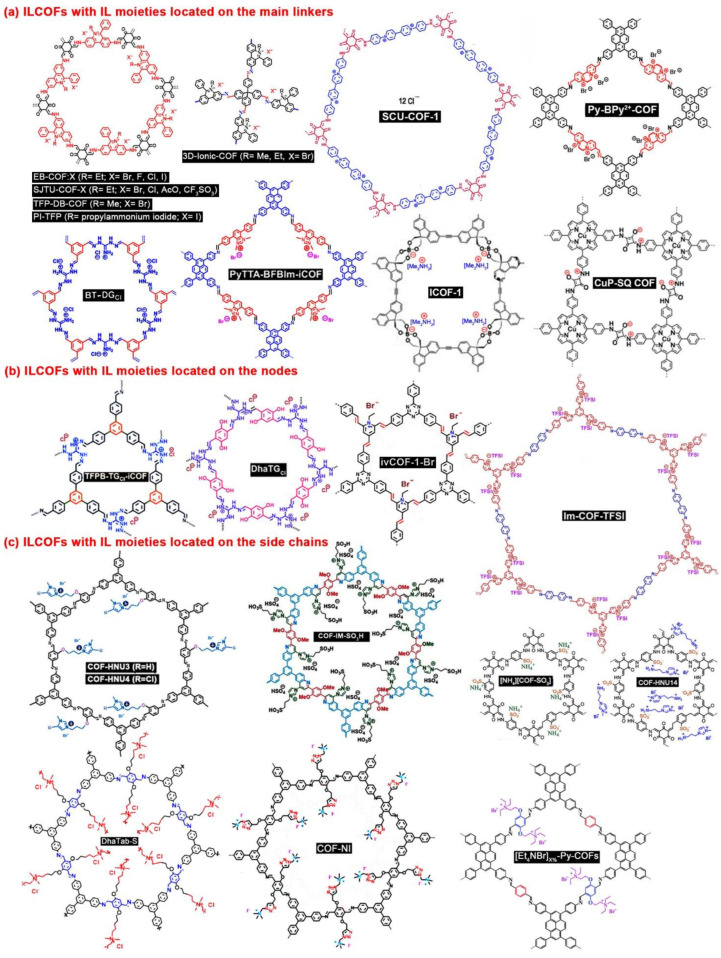
Structures of typical ILCOFs with IL moieties located on (**a**) the main linkers, (**b**) the nodes, and (**c**) the side chains.

**Figure 2:**
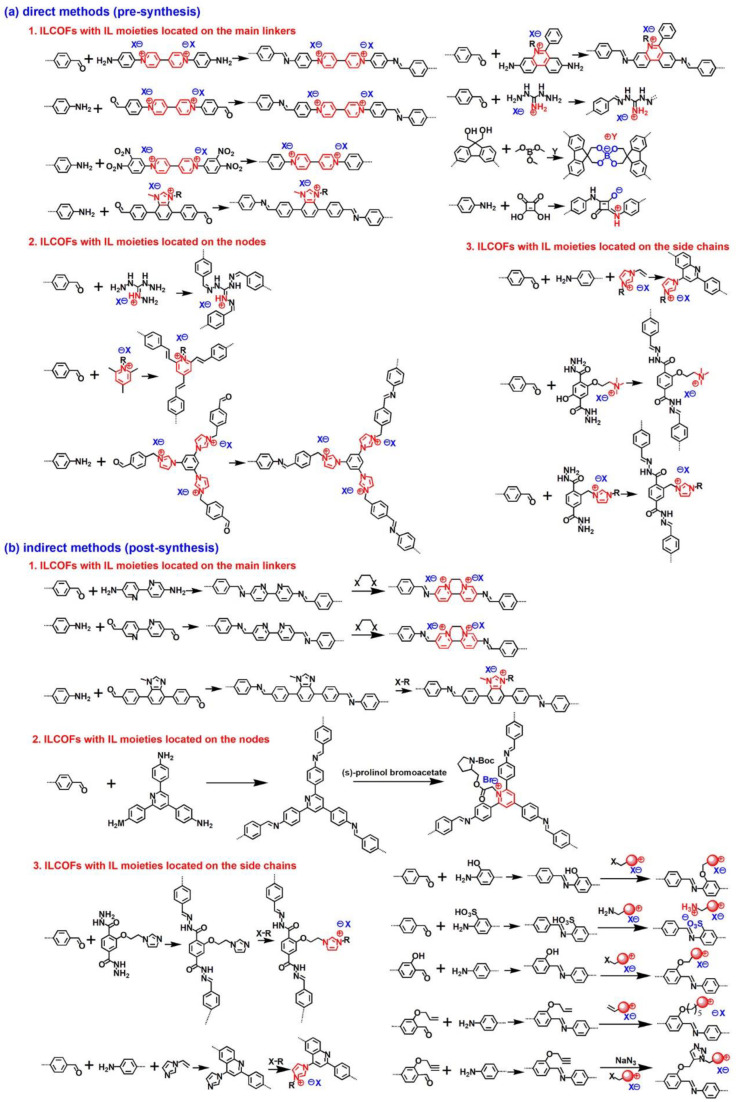
(**a**) Direct methods and (**b**) indirect methods for the synthesis of ILCOFs.

ILCOFs can be classified into three categories according to the locations of IL moieties. The IL moieties can be located on the main linkers, on the nodes, and on the side chains. The structures of different kinds of typical ILCOFs can be found in Figure 1. Because ILs are composed of anions and cations, cations covalently bonded to COFs with free anions are cationic ILCOFs (imidazolium ILCOFs, pyridinium ILCOFs, ammonium ILCOFs, and phosphonium ILCOFs), while anions covalently bonded to COFs with free cations are anionic ILCOFs (spiroborate ILCOFs, squaraine ILCOFs, and sulfonate ILCOFs). Zwitterionic ILCOFs are cations and anions both covalently bonded to COFs. The strategies for the synthesis of ILCOFs can be classified into two categories, including direct methods (pre-synthesis) and indirect methods (post-synthesis) based on the structures of starting materials (Figure 2). As porosity is one of the important properties of COFs, the pore could also be adjusted through tuning the cations and anions of ILs, compared with IL-free COFs, which are only tuned by selecting desired nodes and linkers. Large IL or increased amounts of IL in COFs result in decreased pore sizes.

### 2.1. ILCOFs with IL Moieties Located on the Main Linkers

#### 2.1.1. Cations Are Located on the Main Linkers

##### Pyridinium-linked ILCOFs

**Figure 3:**
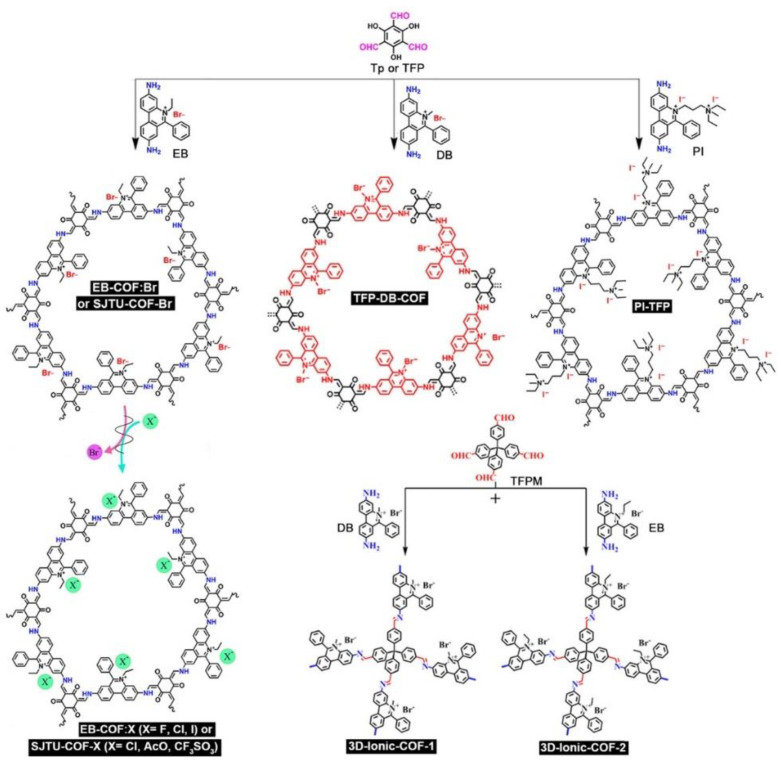
Synthesis of EB-COF:X, SJTU-COF-X, TFP-DB-COF, PI-TFP, and 3D-Ionic-COFs.

Kinds of pyridinium-linked ILCOFs have been developed (Figure 3). Li and Zhang et al. [49] reported the cationic ILCOFs with pyridinium-based cations and halogen anions ([F], [Cl], [Br], and [I]). EB-COF:Br was prepared from 1,3,5-triformylphloroglucinol (Tp) and ethidium bromide (EB), while others were prepared from EB-COF:Br through the anion-exchange method. Thus, dominant pore diameters of 18.4, 17.3, 16.6, and 15.6 Å were obtained. Ajayaghosh et al. [50] reported the supramolecular reassembly of self-exfoliated ionic covalent organic nanosheets from ILCOF (EB-COF:Br) for the label-free detection of double-stranded DNA. Subsequently, Liu, Hua, and Wei et al. [51] prepared a series of 2D ILCOFs, SJTU-COF-X (X=Br, Cl, AcO, CF_3_SO_3_), through the microwave-assisted anion exchange method, and these ILCOFs enhanced CO_2_ capture. More recently, Shi and Wang et al. [52] reported the electrosynthesis of TpEB ILCOF films on nonporous indium-doped tin oxide supports. Zhu and Mi et al. [53] reported a cationic ILCOF (TFP-DB-COF) which was synthesized through imine condensation using Tp as a neutral knot and dimidium bromide (DB) as a cationic linker. Ajayaghosh et al. [54] reported a phenanthridrine-based β-keto enamine-linked ILCOF, PI-TFP, which was synthesized under solvothermal conditions by the condensation reaction of Tp with 3,8-diamino-5-[3-(diethylammonio)propyl]-6-phenanthridinium diiodide (propidium iodide; PI). Fang, Valtchev, and Qiu et al. [55] designed and prepared two porous 3D cationic ILCOFs, 3D-ionic-COF-1 and 3D-ionic-COF-2, through the condensation of tetrahedral tetrakis(4-formylphenyl)methane (TFPM) and linear ionic linkers, DB or EB.

**Figure 4:**
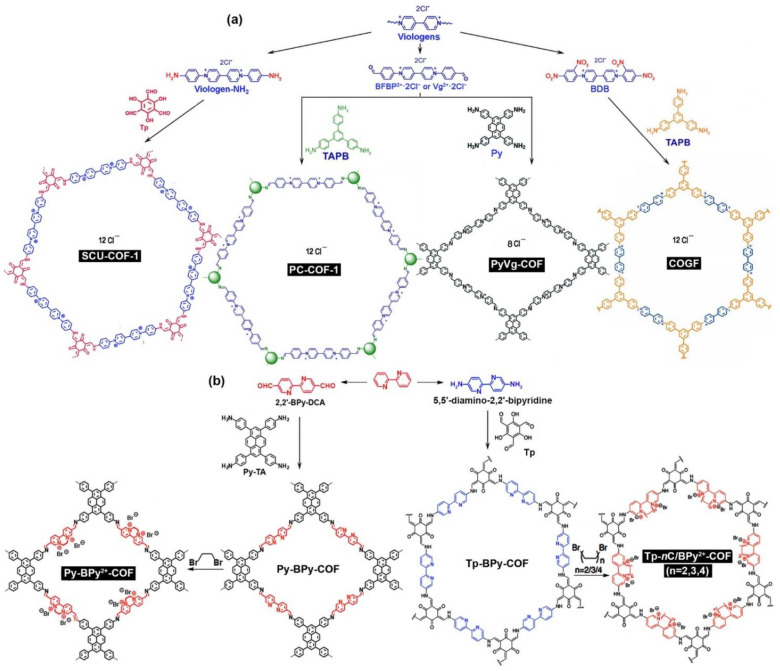
Synthesis of (**a**) viologen-linked ILCOFs (SCU-COF-1, PC-COF-1, PyVg-COF, and COGF) and (**b**) BPy^2+^-linked ILCOFs (Py-BPy^2+^-COF and Tp-nC/BPy^2+^-COF) (*n* = 2,3,4).

Bipyridinium, including 4,4′-bipyridinium and 2,2′-bipyridinium, was used as the linker of ILCOFs (Figure 4). Wang, Liu, and Li et al. [56] prepared polycationic COFs, PC-COF-1 and PC-COF-2, from the condensation of 1,3,5-tris(4-aminophenyl)benzene (Tab or TAPB) and 1,1-bis(4-formylphenyl)-4,4′-bipyridinium dichloride ([BFBP][Cl]_2_) or [BFBP][PF6]_2_. Gu and Ma et al. [57] reported a kind of PyVg-COF via the reticular polycondensation of the knot monomer 4,4′,4″,4‴-(pyrene-1,3,6,8-tetrayl)tetraaniline (Py) with the linker monomer [BFBP][Cl]_2_ under solvothermal conditions. Yang and Wang et al. [58] reported a 2D viologen-based ILCOF, SCU-COF-1, synthesized from an aminated viologen, 1,1-bis(4-aminophenyl)-4,4′-bipyridinium dichloride, as a positively charged linker, with Tp, as a neutral node, via an acid-catalyzed solvothermal synthesis and a procedure of irreversible tautomerism. Trabolsi et al. [59] reported that the form of a viologen-linked covalent organic network could be tuned from amorphous hollow spheres (HS) and tubes (HT) to a crystalline covalent organic gel framework (COGF) using a Zincke reaction between 1,1′-bis(2,4-dinitrophenyl)-[4,4′-bipyridine]-1,1′-diium dichloride (BDB) and an aromatic amine, TAPB, under either solvothermal or microwave conditions.

Yang and Guo et al. [60] converted a 2,2′-bipyridine-based COF from neutral Py-BPy-COF to positively charged Py-BPy^2+^-COF and, ultimately, to a cationic radical framework, Py-BPy^+•^-COF, through sequential in situ reactions, quaternization, and one-electron reduction. The cationic radical framework enabled the superimposition of redox centers with each other in the framework. Sun and Zhang et al. [61] reported four highly porous metalloporphyrin-based ILCOFs for CO_2_ cycloaddition. These ILCOFs were synthesized by coupling a nitro-containing porphyrin, 5,10,15,20-tetrakis(4-nitrophenyl)porphyrin (TNPP), with one of the following diamino compounds: 3,8-diamino-6-phenylphenanithridine (NPPN) or 5,5′-diamino-2,2′-bipyridine (NBPy). This was followed by ionization/quaternization with bromoethane (C_2_H_5_Br) or dibromoethane (C_2_H_4_Br_2_) and then metalization with Zn or Co. Guo et al. [62] reported a series of 2,2′-bipyridinium ILCOFs, Tp-nC/BPy^2+^-COF (*n* = 2,3,4), for photocatalytic H_2_ evolution from water splitting. These ILCOFs were synthesized from Tp-BPy-COF by a controllable post-quaternization reaction, and the relative molar ratios of cyclic diquats to 2,2′-bipyridine moieties in Tp-nC/BPy^2+^-COF largely depended on the reaction time. When Tp-2C/BPy^2+^-COF was synthesized through the direct method, the product would become amorphous with the increasing amount of 2C/BPy^2+^.

##### Imidazolium-linked ILCOFs

**Figure 5:**
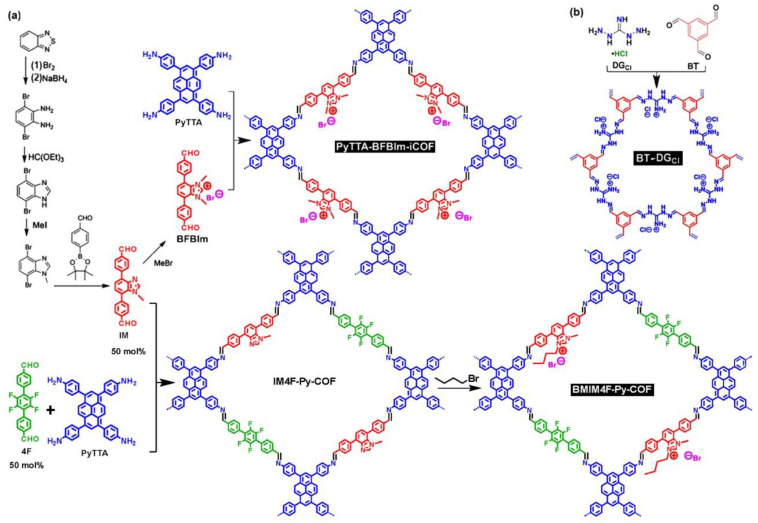
Synthesis of (**a**) benzimidazolium-linked ILCOFs and (**b**) diaminoguanidinium ILCOF.

There are some examples of benzimidazolium-linked ILCOFs (Figure 5a). Jiang et al. [63] synthesized 4,4′,4′′,4′′′-(pyrene-1,3,6,8-tetrayl) tetraaniline (PyTTA) as a neutral knot and 5,6-bis(4-formylbenzyl)-1,3-dimethyl-benzimidazolium bromide (BFBIm) as a cationic linker for the construction of imine-linked positively charged COFs, PyTTA-BFBIm-iCOF, in which the benzimidazolium cationic sites were exposed to the wall surface. Zeng, Xu, and Gao et al. [52] reported an imine-linked ILCOF, BMIM4F-Py-COF, post-synthesized from IM4F-Py-COF. IM4F-Py-COF was prepared via a three-component condensation reaction of PyTTA, 4,7-bis(4-formylphenyl)-1-methyl-1H-benzimidazole (IM), and 2′3′5′6′-tetrafluoro-[1,1′:4′,1′′-terphenyl]-4,4′-dicarbaldehyde (4F).

##### Guanidinium-linked ILCOFs

A guanidinium-based ILCOF, BT-DGCl, was reported by Jansone-Popova et al. [64] and synthesized from benzene-1,3,5-triscarbaldehyde (BT) and diaminoguanidine hydrochloride (DG_Cl_) (Figure 5b). Their PXRD analysis revealed the low crystallinity of BT-DG_Cl_ due to the presence of repulsive interactions between the positively charged guanidinium groups combined with the necessity to accommodate chloride counterions. These ILCOFs were used for the rapid and selective removal of toxic Cr(VI) oxoanions from water. Jia et al. [65] reported the magnetic Fe_3_O_4_@ BT-DG_Cl_ for phosphopeptides capture.

#### 2.1.2. Anions Are Located on the Main Linkers

**Figure 6:**
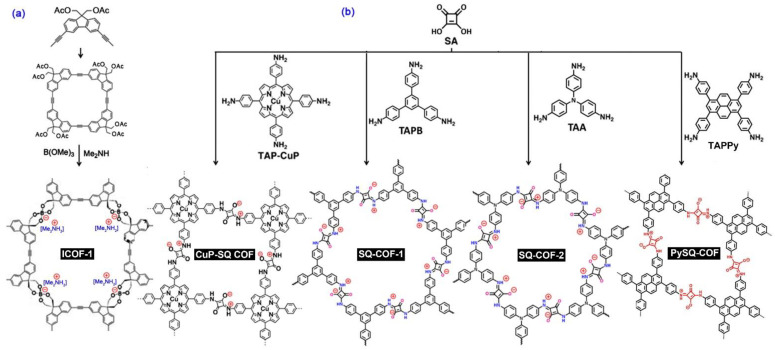
Synthesis of (**a**) spiroborate-linked ILCOF (ICOF-1) and (**b**) squaraine-linked ILCOFs (CuP-SQ-COF, SQ-COF-1, SQ-COF-2, and PySQ-COF).

There are two kinds of anionic ILCOFs with anions located on the main linkers, including the spiroborate anion and squaraine anion (Figure 6). 

##### Spiroborate-linked ILCOFs

Lee and Zhang et al. [66] synthesized two ICOFs with spiroborate linkage. ICOF-1 with [Me_2_NH_2_]^+^ was prepared from a macrocycle molecule with B(OMe)_3_ and Me_2_NH. By using LiOH as the base instead of Me_2_NH, ICOF-2 was obtained. Feng et al. [67] reported a series of 3D anionic cyclodextrin (CD)-based COFs through the condensation of γ-CD and B(OMe)_3_. When the reaction was in the presence of LiOH under microwave-assisted solvothermal conditions, CD-COF-Li was obtained (Li^+^ is the counterion). When the proton acceptor in the reaction was changed to dimethylamine (DMA) or piperazine (PPZ), CD-COF-DMA ([HDMA]^+^ is the counterion) and CD-COF-PPZ ([H_2_PPZ]^2+^ is the counterion) were obtained. It is obvious that CD-COF-DMA and CD-COF-PPZ are ILCOFs. Owing to the high porosity, flexible building blocks, and charged skeleton, CD-COFs show great potential in the fields of ion conduction and gas separation. Subsequently, Li and Zhang et al. [68] theoretically investigated the topology of spiroborate-linked ILCOFs.

##### Squaraine-linked ILCOFs

Squaraines (SQs), prepared through the condensation of squaric acid (SA) with aromatic compounds, are a class of organic dyes with a unique resonance-stabilized zwitterionic structure. Only a few examples of COFs have been explored. For example, Jiang et al. [69] reported a crystalline SQ-linked 2D conjugated CuP-SQ COF with a zwitterionic structure. CuP-SQ COF was synthesized from the condensation of SA and copper(II) 5,10,15,20-tetrakis(4-aminophenyl)porphyrin (TAP-CuP). Recently, Fan and Zhang et al. [70] reported two photoactive SQ-linked COFs (SQ-COF-1 and SQ-COF-2), prepared through the condensation of SA with TAPB or tris(4-aminophenyl)amine (TAA). Guo et al. [71] investigated the multivariate synthesis of SQ-linked COFs, Py-TPA_1-X_-SQ_X_ (X = [SA]/([TPA] + [SA])), by the reaction of 4,4′,4″,4‴-(pyrene-1,3,6,8-tetrayl) tetraaniline (TAPPy) with terephthalaldehyde (TPA) and SA. TPA was a neutral structural stabilizer. When X = 85~90%, a fully SQ-linked COF (PySQ-COF) was formed, while, when X ≥ 95%, the resulting polymer was amorphous in structure.

### 2.2. ILCOFs with IL Moieties Located on the Nodes

#### 2.2.1. Guanidinium-noded ILCOFs

**Figure 7:**
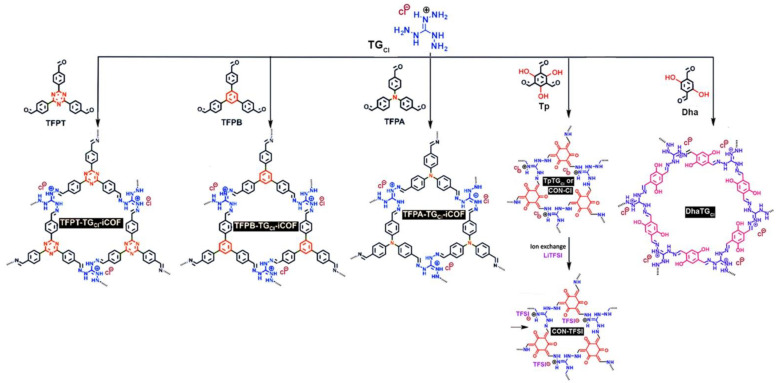
Synthesis of TFPT-TG_Cl_-iCOF, TFPB-TG_Cl_-iCOF, TFPA-TG_Cl_-iCOF, CON-Cl, CON-TFSI, and DhaTG_Cl_.

Due to their C3 symmetric property, triaminoguanidinium halides (TG_Cl_, TG_Br_, and TG_I_) have been used as nodes for designing ILCOFs (Figure 7). Banerjee et al. [72] prepared three 2D guanidinium-based ionic covalent organic framework nanosheets (iCONs) (TpTG_Cl_, TpTG_Br_, and TpTG_I_) through C3 symmetric Tp reacting with the synthesized TG_Cl_, TG_Br_, and TG_I_, respectively. These iCONs displayed stable aqueous dispersion for at least 20 days, owing to their ionic backbone. Chen and Chen et al. [73] reported cationic CONs for fast Li-ion conduction. They first synthesized cationic CON with chloride counterions (CON-Cl), and then the CON-Cl was ion-exchanged with LiTFSI to form CON-TFSI. The XPS full-spectrum scans showed that the Cl signal almost disappeared after ion exchange. Yan et al. [74] reported the design and synthesis of an iCON (DhaTG_Cl_) from 2,5-dihydroxyterephthalaldehyde (Dha) and triaminoguanidinium chloride (TG_Cl_) via the Schiff reaction for the rapid and selective trapping of ReO_4_^–^ anions. They also reported other guanidinium-noded ILCOFs, including TFPT-TG_Cl_-iCOF, TFPB-TG_Cl_-iCOF, and TFPA-TG_Cl_-iCOF, which could be prepared from TG_Cl_ and 2,4,6-Tris(4-formylphenyl)-1,3,5-triazine (TFPT), 1,3,5-Tris(4-formylphenyl) benzene (TFPB), or tris(4-formylphenyl) amide (TFPA), respectively [75]. Sarkar and Pal et al. [76] reported the proton-triggered fluorescence switching in self-exfoliated DhaTG_Cl_ for applications in the selective detection of anions ([F]^–^, [Br]^–^, [I]^–^, [NO_3_]^–^, [HPO_4_]^–^, [HSO_4_]^–^, and [SCN]^–^). Recently, Wu, Guiver, and Jiang et al. [77] reported the oil–water–oil triphase synthesis of DhaTG_Cl_ iCONs.

#### 2.2.2. Pyridinium-noded ILCOFs

**Figure 8:**
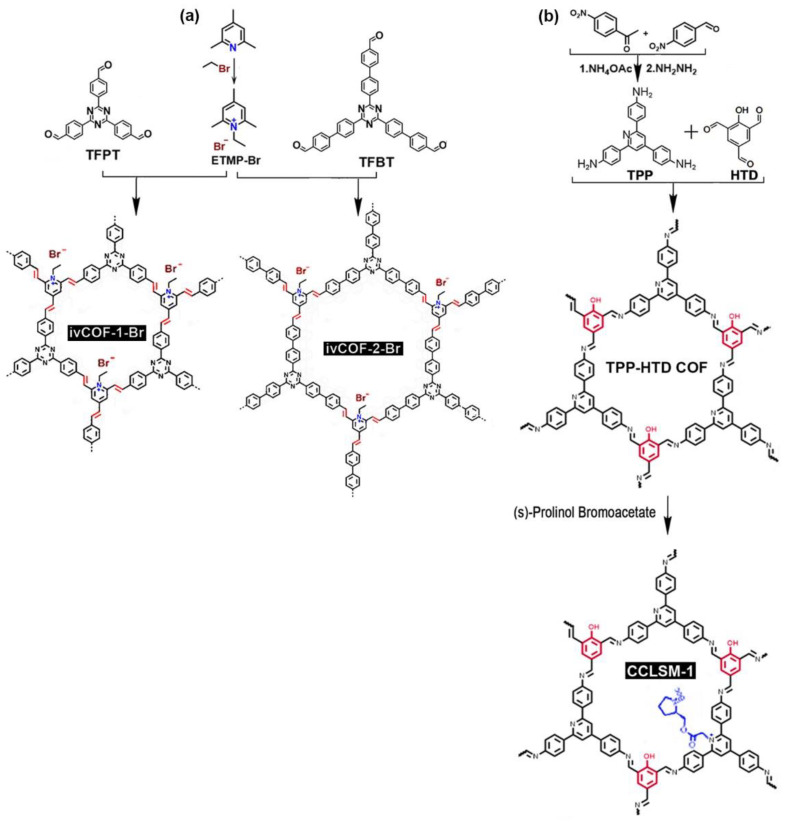
Synthesis of (**a**) ivCOF-1-Br, ivCOF-2-Br, and (**b**) CCLSM-1.

Zhang et al. [78] reported the synthesis of 2D pyridinium-based ionic vinylene-linked COFs (ivCOF-1-Br and ivCOF-2-Br) by reticulating N-ethyl-2,4,6-trimethylpyridinium halide ([ETMP][Br] or [ETMP][I]) with multi-topic aromatic aldehyde derivatives, TFPT and 1,3,5-tris-(4′-formyl-biphenyl-4-yl)triazine (TFBT), through a quaternization-promoted Knoevenagel condensation. These ILCOFs exhibited large surface areas (1343 m^2^ g^−1^) and regular open channels with diameters centered at 1.4 nm and 1.9 nm. Zhang et al. [79] reported another pyridinium chiral ILCOF as a catalyst for asymmetric Henry reactions. They first synthesized TPP-HTD COF, starting from TPP and HTD via imine formation, and then chiral ILCOF, CCLSM-1, was prepared by the post-modification of TPP-HTD COF with (S)-prolinol bromoacetate. The structures and synthesis of pyridinium-noded ILCOFs can be found in Figure 8.

#### 2.2.3. Imidazolium-noded ILCOFs

**Figure 9:**
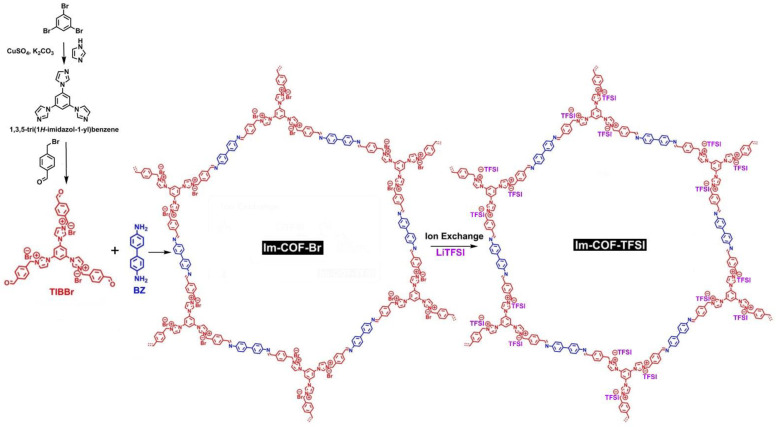
Synthesis of imidazolium-linked ILCOFs (Im-COF-Br and Im-COF-TFSI).

An imidazolium ILCOF, Im-COF-Br, was prepared by Ding, Han, and Feng et al. [80] via a [3 + 2] condensation reaction under solvothermal conditions using 1,3,5-tris [3-(4-formylbenzyl)-1H-imidazol-1-yl]benzene bromide (TIBBr) as the cationic building block and benzidine (BZ) as the neutral unit. By adopting the ion-exchange strategy, the counter-ion [Br]^–^ was replaced with [TFSI]^–^, and Im-COF-TFSI improved the lithium-ion conductivity (Figure 9).

### 2.3. ILCOFs with IL Moieties Located on the Side Chains

#### 2.3.1. Cations Are Located on the Side Chains

##### Imidazolium-grafted ILCOFs

**Figure 10:**
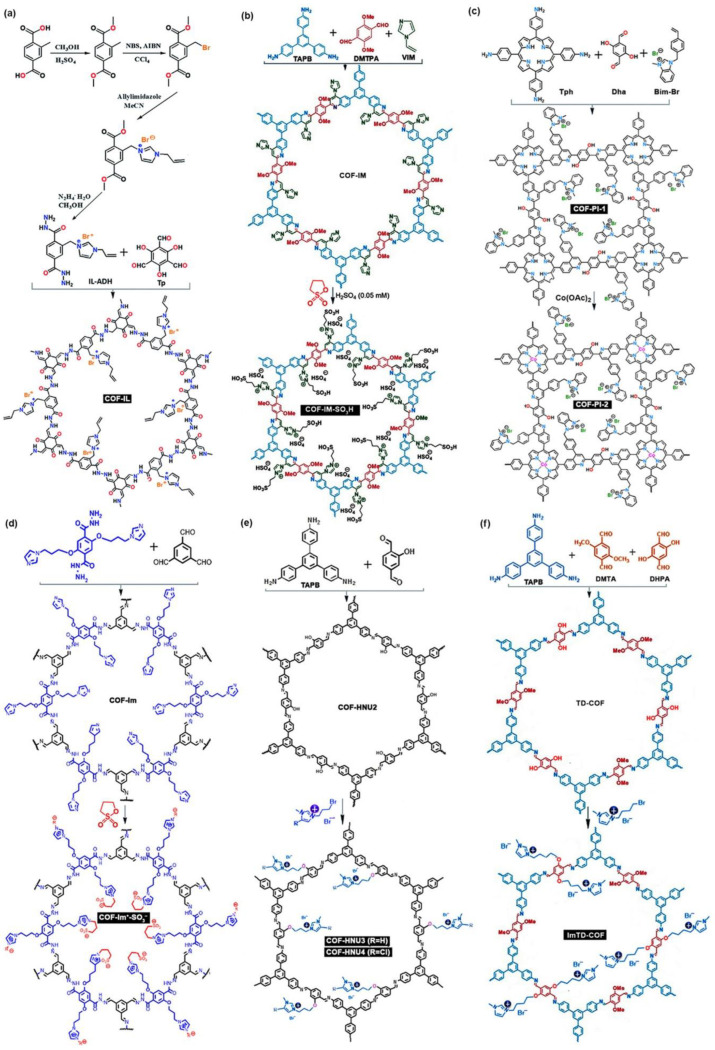
Synthesis of (**a**) COF-IL, (**b**) COF-IM-SO_3_H, (**c**) COF-PI-1 and COF-PI-2, (**d**) COF-Im^+^-SO_3_^–^, (**e**) COF-HNU3 and COF-HNU4, and (**f**) ImTD-COF.

Yao and Dong et al. [81] synthesized a COF-IL by the reaction of Tp with allyl-imidazolium-based IL-functionalized terephthalohydrazide (IL-ADH). Subsequently, the same authors reported a kind of sulfonic acid functionalized quinoline-linked imidazolium ILCOF, COF-IM-SO_3_H, by the three-component one-pot Povarov reaction and post-synthetic modification [82]. They also reported other quinoline-linked porphyrin-containing ILCOFs (COF-PI-1 and COF-PI-2) with or without metal coordination [83]. COF-PI-1 was prepared from amino-substituted porphyrin 5,10,15,20-tetrakis(4-aminophenyl) porphyrin (Tph), imidazolium-IL-attached styrene 3-methyl-1-(4-vinylbenzyl)-benzimidazolium bromide (Bim-Br), and Dha, and then COF-PI-2 was obtained via the post-synthetic metallization of the porphyrin entity by Co(OAc)_2_ in COF-PI-1. Wang, Xu, and Zhang et al. [84] synthesized a hydrazone-linked ILCOF, COF-Im^+^-SO_3_^–^, from 1,3-propane sultone and COF-Im, which was prepared from 1,3,5-trisformylbenzene and 2,5-bis(3-(1H-imidazole-1-yl) propoxy)terephthalohydrazide. Wang and Wang et al. [85] reported two imidazolium ILCOFs (COF-HNU3 and COF-HNU4) via the Williamson reaction of 1-(3-bromopropyl)-3-methyl-1H-imidazol-3-ium bromide or 1-(3-bromopropyl)-4-chlorine-3-methyl-1H-imidazol-3-ium bromide with COF-HNU2, which was synthesized by the Schiff reaction of 2-hydroxybenzene-1,4-dialdehyde and TAPB. Yang, Qiao, and Han et al. [86] reported an imine-linked imidazolium-based ImTD-COF through the Schiff reaction of TAPB, 2,5-dihydroxyterephthalaldehyde (DHPA or DHTA), and 2,5-dimethoxyterephthalaldehyde (DMTA) and a subsequent Williamson reaction with [BMIm]Br. Through the ion-exchange process, three [Br]^–^ anions in the ImTD-COF could be replaced with one [PW_12_O_40_]^3–^ anion to obtain POM@ImTD-COF. Zhang, Xu, and Qiao et al. [87] reported an ILCOF, IL-COFTAPB-DHPA, via imidazolium IL post-grafted inside the COFTAPB-DHPA pore channel, increasing the effective surface area and maintaining the rapid ion transport. Figure 10 shows the structures of these imidazolium-grafted ILCOFs.

##### Ammonium-grafted ILCOFs

Several ammonium-grafted ILCOFs were synthesized via three-component condensation. In 2016, Gao et al. [88] reported the post-synthesis of [Et_4_NBr]_X%_-Py-COFs (X = 25, 50, 75, 100) with the ionization of channel walls through the Williamson ether reaction of (2-bromoethyl)triethylammonium bromide with [HO]_X%_-Py-COFs, which were synthesized by the condensation of 4,4′,4″,4‴-(pyrene-1,3,6,8-tetrayl) tetraaniline (PyTTA) with DHPA and terephthalaldehyde (PA) at various molar ratios. The results indicated the crystal structures of [Et_4_NBr]_X%_-Py-COFs (X = 25, 50) and the amorphous frameworks of [Et_4_NBr]_X%_-Py-PAFs (X = 75, 100). Similarly, Ding, Chen, and Han et al. [89] prepared several zwitterionic ILCOFs, [BE]_X%_-TD-COFs, by introducing betaine groups (BE) onto the channel walls of pre-synthesized imine-linked COFs via pore surface engineering methodology. Recently, Han et al. [90] post-synthesized a series of iCOF-AB-Xs (X = 33, 50, 67, and 100) through the Williamson ether reaction of initial COF-OH-X with (2-bromoethyl)triethylammonium bromide (AB). The imine-linked COF-OH-X COFs were prepared from 2,4,6-tri(4-aminophenyl)-1,3,5-triazine (TAPT), DMTA, and DHTA, and X represented the molar percentage of DHTA to DHTA + DMTA. Xu and Zhang et al. [91] reported a series of polymerized ILCOFs, PIL-COF-X (X = 0.33, 0.5, 1.0). COF-V-Xs (X = 0.33, 0.5, 1.0) were prepared from TAPB, 2,5-divinylterephthalaldehyde (DVTP), and 2,5-dimethoxyterephthalaldehyde (DMTP). X was the mole ratio of DVTP to DVTP + DMTP. Then, PILCOFs were prepared via a consequent copolymerization and quaternization of COF-V-X. With a proper X value, the resultant PIL-COF-X still retained the crystallinity and porosity.

**Figure 11:**
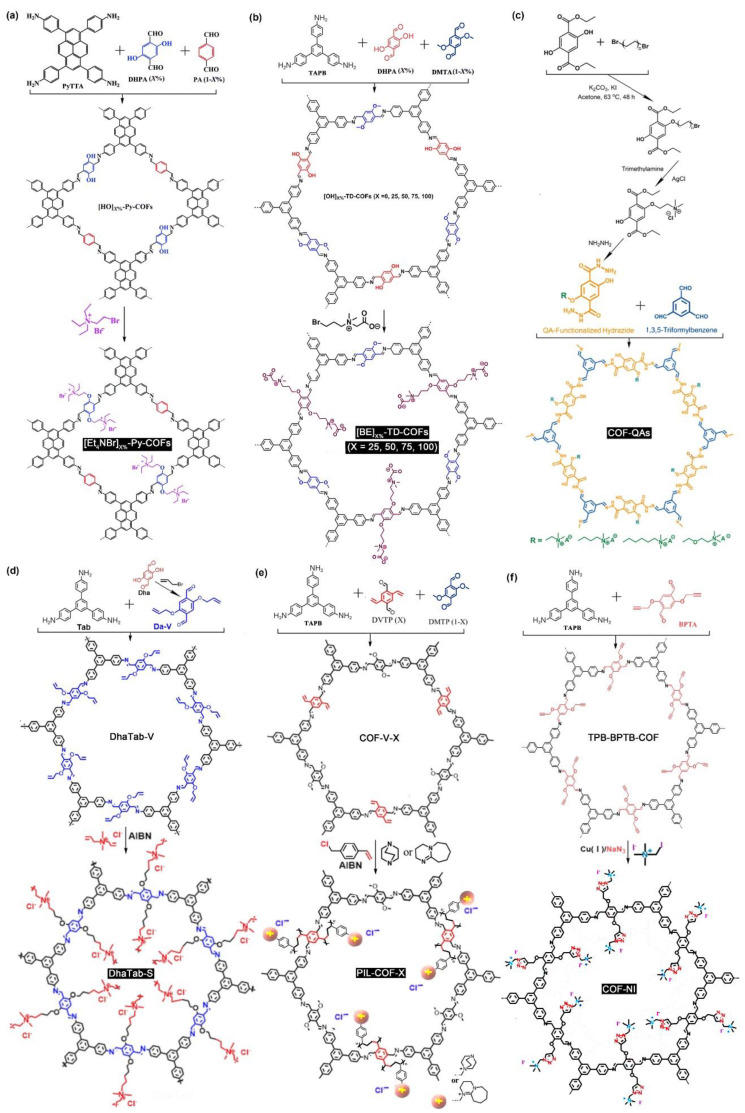
Synthesis of ammonium-grafted ILCOFs: (**a**) [Et_4_NBr]_X%_-Py-COFs, (**b**) [BE]_X%_-TD-COFs (X = 25, 50, 75, 100), (**c**) COF-QAs, (**d**) DhaTab-S, (**e**) PIL-COF-X, and (**f**) COF-NI.

Different from three-component condensation, which should control the X value to retain the crystallinity and porosity, ammonium-grafted ILCOFs synthesized via two-component condensation were always obtained with crystallinity. Li and Liao et al. [92] reported a europium (Eu)-containing ILCOF (DhaTab-COF-EuIL) as a sensitive and selective acetone sensor. This ILCOF was microporous and crystalline and synthesized via a Schiff-base reaction between Dha and Tab, followed by an IL-modification (Williamson ether reaction) with AB and then an ion displacement with a Eu-based chelate anion. Yan et al. [93] prepared a kind of cationic ILCOF, DhaTab-S, via free-radical polymerization between a cationic surfactant, diallyldimethylammonium chloride (DMDAAC), and a vinyl-containing COF, DhaTab-V, which was prepared from Tab and vinyl-modified 2,5-dihydroxyterephthalaldehyde (Da-V). More recently, Liang and Qiu et al. [94] prepared a kind of ILCOF, Tp-BDOH-AB, through Williamson ether reactions between Tp-BDOH and AB, and Tp-BDOH COF was synthesized via the Schiff reaction of Tp and 3,3′-dihydroxybenzidine (BDOH). This ILCOF was studied for the efficient detection and adsorption of ReO_4_^–^/TcO_4_^–^. Jiang et al. [95] reported a series of quaternary ammonium (QA) functionalized nanoplate-like COF-QAs by the reaction of hydrazide building units with aldehyde units. Sui, Tian, and Chen et al. [96] reported a mesoporous cationic ILCOF (COF-NI) prepared by post-grafting the quaternary ammonium salt group into the pore channel of TPB-BPTP-COF. The one-pot post-synthesis was performed in dry DMF using CuI as the catalyst to react iodomethyltrimethylammonium iodide and NaN_3_ with TPB-BPTP-COF. The structures and synthesis of typical ammonium-grafted ILCOFs can be found in Figure 11.

##### Phosphonium-grafted ILCOFs

There is only one example. Ding, Chen, and Han et al. [97] reported [PTPP]_X%_-TD-COFs (X = 25, 50, 75) for versatilely catalyzing the chemical transformations of CO_2_. X represented the molar percentage of DHTA to DHTA + DMTA. The synthesis of [PTPP]_X%_-TD-COFs was similar to that of [Et_4_NBr]_X%_-Py-COFs and [BE]_X%_-TD-COFs.

#### 2.3.2. Anions Are Located on the Side Chains

**Figure 12:**
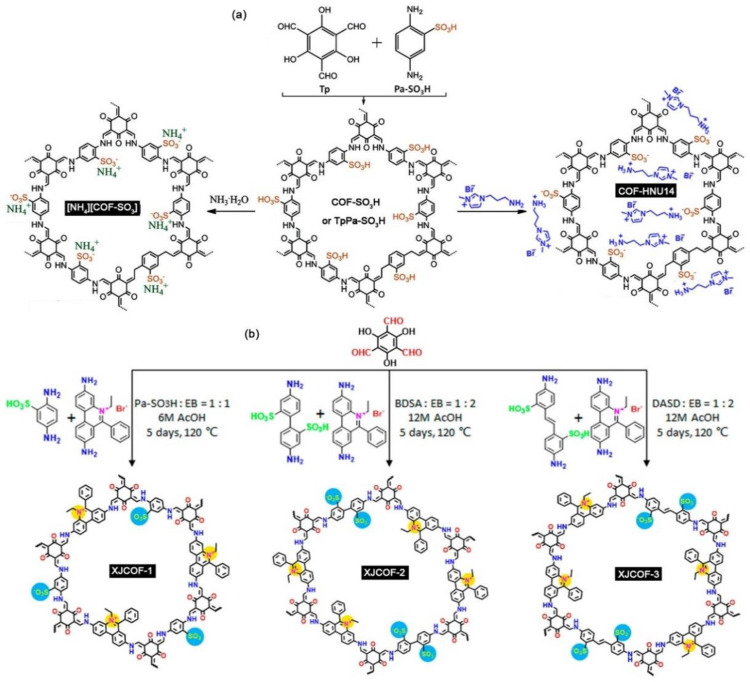
Synthesis of (**a**) [NH_4_][COF-SO_3_] and COF-HNU14 and (**b**) zwitterionic XJCOFs. Reprinted with permission from ref. [100]. Copyright 2021 American Chemical Society.

ILCOFs with sulfonate anions located on the side chains were reported (Figure 12). Luo et al. [98] reported an ammoniating COF for the extraction of uranium ions (UO_2_^2+^). They first prepared the SO_3_H-anchored COF (COF-SO_3_H), and then the ion-exchange material of [NH_4_][COF-SO_3_] was obtained by immerging COF-SO_3_H in NH_3_·H_2_O. Such material ([NH_4_][COF-SO_3_]) also contained abundant -SO_3_^−^ units in the pore wall that could implement the coordination interaction toward uranyl. Qiu and Wang et al. [99] reported a kind of ILCOF, COF-HNU14, for CO_2_ fixation. They first prepared the SO_3_H-anchored COF (TpPa-SO_3_H) from Tp and 2,5-diaminobenzenesulfonic acid (Pa-SO_3_H). TpPa-SO_3_H contained a large number of Bronsted acid sites and could implement the coordination interaction toward the basic IL 1-aminopropyl-3-methylimidazolium bromide ([APMIm][Br]) to form COF-HNU14. Ma et al. [100] reported three zwitterionic ILCOFs (XJCOF-1, XJCOF-2, XJCOF-3) containing equal numbers of anionic sulfonate and cationic ethidium groups. These ILCOFs were prepared from three sulfonate-containing anilines as anionic monomers and EB as cationic monomers through the Schiff reaction and the subsequent washing process to remove the hydrogen ion and the bromide ion.

## 3. CO_2_ Conversion by ILCOFs

With the help of ILCOFs as sorbents and catalysts, CO_2_ can be captured and converted into kinds of value-added chemicals through different reactions, such as the reduction of CO_2_ with amine, the cycloaddition of CO_2_ with propylene oxide, etc.

### 3.1. CO_2_ Capture

CO_2_ capture is an important topic in chemistry and a sustainable world. ILCOFs have a potential adsorption performance for CO_2_ capture due to their porous structures and active sites. For example, Gao et al. [88] reported that an ILCOF could be used for CO_2_ capture, and the capacity was 164.6 mg CO_2_ per g IL at 0 °C and 1 bar. Dong et al. [81] synthesized an ILCOF with a highly selective adsorption for CO_2_ over CH_4_, N_2_, and H_2_ due to the relatively large porosity and the high density of imidazolium-based IL groups in ILCOF. Their results showed a CO_2_ uptake amount of 106.04 cm^3^ g^−1^ at 0 °C and 59.37 cm^3^ g^−1^ at 25 °C under 1 bar, respectively. Only small uptake amounts of CH_4_ (19.15 cm^3^ g^−1^ at 0 °C, and 11.88 cm^3^ g^−1^ at 25 °C), N_2_ (7.29 cm^3^ g^−1^ at 0 °C, and 5.24 cm^3^ g^−1^ at 25 °C), and H_2_ (1.36 cm^3^ g^−1^ at 0 °C, and 0.78 cm^3^ g^−1^ at 25 °C) were observed for ILCOF under the same conditions. Subsequently, Liu, Hua, and Wei et al. [51] showed that SJTU-COF-X (X=Br, Cl, AcO, CF_3_SO_3_) enhanced CO_2_ capture. Among them, the acetate anion containing ILCOF showed a CO_2_ capacity of 171 mg g^−1^ at 0 °C and under 1 bar, which was increased to 1.7 times compared with that of the pristine COF.

### 3.2. Reduction of CO_2_ with Amine

**Figure 13:**
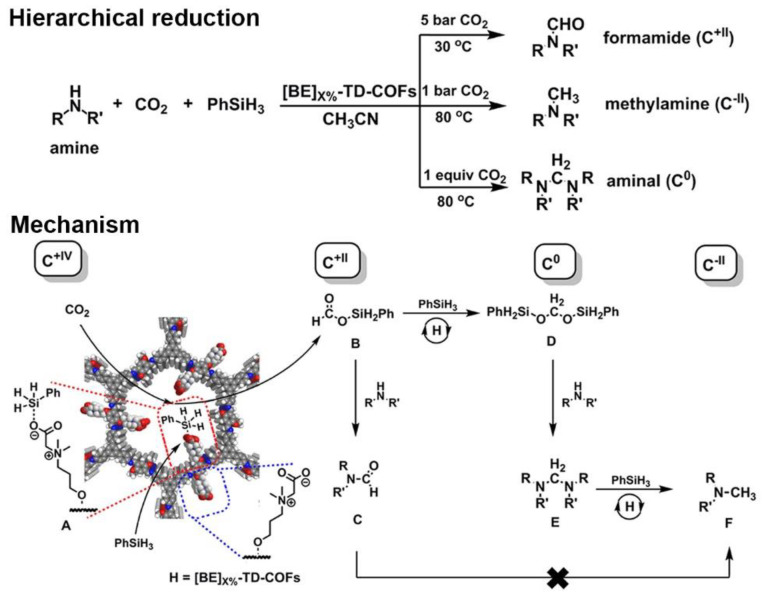
Hierarchical reduction of CO_2_ with amine and PhSiH_3_ to afford formamide, methylamine, and aminal. Reprinted with permission from ref. [89]. Copyright 2018 American Chemical Society.

Amine could be formylated through the hierarchical reduction of CO_2_ with amine as a substrate under phenylsilane (PhSiH_3_) as a reductant, and different kinds of products (formamides, methylamines, and aminals) will be obtained through different pathways [101]. Gao et al. [88] showed that, with the amount of 5 mol% [Et_4_NBr]_50%_-Py-COF as the catalyst, the N-methylformanilide with an isolated yield of 94% could be obtained through the formylation at 30 °C in DMF as the solvent, and the molar ratio of amine to PhSiH_3_ was 1:2. Their results suggested that the [Et_4_NBr]_50%_-Py-COF behaved as a bifunctional catalyst, which activated PhSiH_3_ to react with CO_2_, yielding formoxysilane, and activated the amine through the hydrogen bond. As the carboxylate of betaine could activate CO_2_ and enhance the reducibility of PhSiH_3_ [102], Han et al. reported several zwitterionic ILCOFs, [BE]_X%_-TD-COFs (X = 25, 50, 75, 100) [89], and quaternary phosphonium ILCOFs, [PTPP]_X%-_TD-COFs (X = 25, 50, 75) [97], for the hierarchical reduction of CO_2_. Their results showed that [BE]_50%_-TD-COF and [PTPP]_50%_-TD-COF possessed the highest catalytic activity. The general mechanism showed that active hydride could be transferred from the hypervalent silicon species A to CO_2_ to generate silyl formate B, which could be further reduced to generate silyl acetal D. B and D could continuously react with amines to achieve formamide C and aminal E, and E could be ultimately converted into the methylated product F. Furthermore, high CO_2_ pressure resulted in product C, while low CO_2_ pressure resulted in product E under certain temperatures (Figure 13). Wang and Wang et al. [85] studied the formylation of various amines with CO_2_ and PhSiH_3_ using COF-HNU3 as an efficient catalyst. The mesoporosity and ordered open channels of COF-HNU3 contributed to the exposed active sites, favored the fast transportation of the substrates, and promoted the rapid conversion of the reactants.

### 3.3. Cycloaddition of CO_2_ with Epoxides

**Figure 14:**
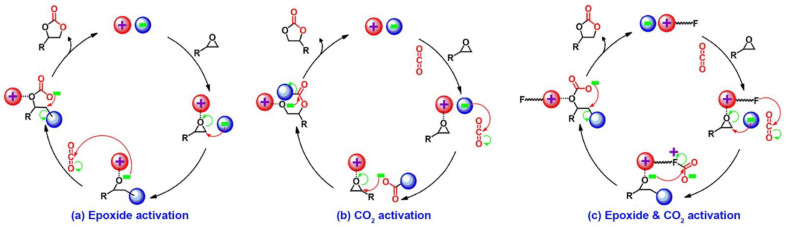
Mechanisms for CO_2_ cycloaddition. (**a**) Epoxide activation; (**b**) CO_2_ activation; (**c**) epoxide and CO_2_ activation.

The cycloaddition of CO_2_ and epoxides to form value-added cyclic carbonates is a 100% atom-economical reaction and one of the efficient routes for CO_2_ chemical fixation [103]. It is known that ILs or polymeric ILs with active hydrogen atoms or hydroxyl groups and halides or others will result in the efficient cycloaddition of CO_2_ with epoxides [104,105,106,107,108,109,110,111]. The proposed mechanisms can be classified into three pathways, including the “epoxide activation” pathway, “CO_2_ activation” pathway, and “epoxide & CO_2_ simultaneous activation” pathway (Figure 14).

It was reported that the mechanism of the catalytic CO_2_ cycloaddition over ILCOFs was similar to that over ILs. Yao and Dong et al. [81] reported an IL-decorated COF, COF-IL, which could be used as a highly active catalyst for CO_2_ cycloaddition with epoxides under mild conditions (1 atm and ≤80 °C), without any co-catalyst assistance. They found that the epoxide was activated by COF-IL through the interaction of C2-H on the imidazolium with the oxygen on the epoxide. The results indicated that the catalytic performance of COF-IL exhibited a positive correlation, with increases in the reaction temperature, catalyst amount, and reaction time. Subsequently, as metalloporphyrin-containing materials usually possess visible-light-induced photothermal conversion behavior [112], the same authors also reported two quinoline-linked porphyrin-containing ILCOFs with or without metal coordination for catalytic CO_2_ cycloaddition via visible-light-induced photothermal conversion [83]. Sun and Zhang et al. [61] reported four highly porous metalloporphyrin-based ILCOFs for highly efficient CO_2_ cycloaddition. They showed that the metal sites contacted with the oxygen on the epoxide, resulting in the activation of the epoxide as well as the formation of an M-O bond. Wang et al. [85,99] synthesized two cationic ILCOFs (COF-HNU3 and COF-HNU4) and an anionic ILCOF (COF-HNU14) for the highly efficient catalysis of CO_2_ cycloaddition with different epoxides under solvent-free and co-catalyst-free conditions, owing to the excellent porosity and high density of the active sites of imidazolium salts within the nanoscopic channels of ILCOFs. In these IL-functionalized COFs, the turnover number (TON) of COF-HNU3 was as high as 495000. Yang, Qiao, and Han et al. [86] reported that imidazolium-based IL-decorated COFs with the [PW_12_O_40_]^3–^ anion (POM@ImTD-COF) showed high catalytic activity for CO_2_ cycloaddition reaction under mild conditions (1 bar and 80 °C), with an IL as the co-catalyst ([N_4444_][Br]).

## 4. Conclusions and Outlook

**Figure 15:**
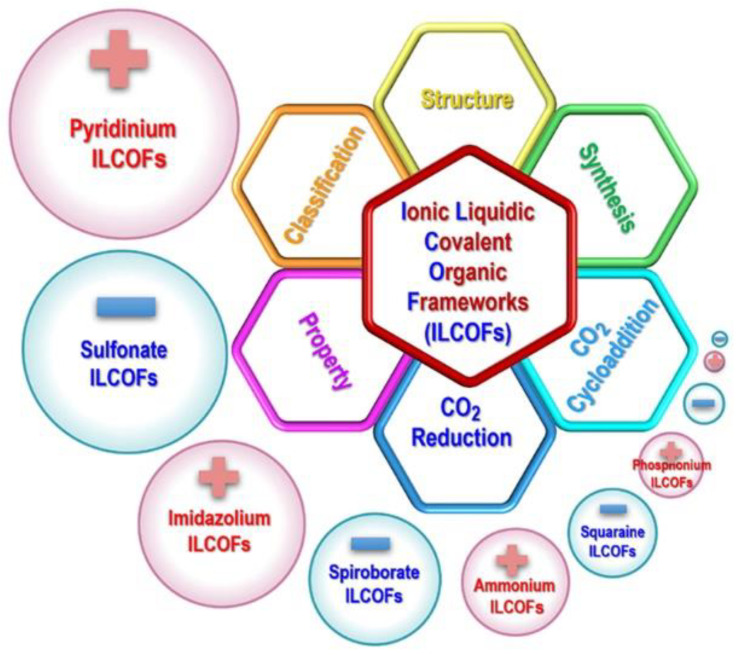
A summary on different kinds of ILCOFs and related topics.

With the combination of unique structures and properties of ionic liquids (ILs) and covalent organic frameworks (COFs), covalent organic frameworks with ionic liquid-moieties (ILCOFs) have been developed as a kind of novel and efficient sorbent, catalyst, and electrolyte since 2016. In this critical review, we first focus on the structures and synthesis of different kinds of ILCOFs materials, including ILCOFs with IL moieties located on the main linkers, on the nodes, and on the side chains. We then discuss the ILCOFs for CO_2_ conversion, including the reduction and cycloaddition of CO_2_ (Figure 15). It is clear that the field of ILCOFs is still in its infancy.

Several issues should be given more attention and need to be investigated further. For example, functionalized ILs strategies are useful for designing efficient CO_2_-philic ILCOFs. It is known that the functionalized ILs containing active sites on cations or anions are efficient for CO_2_ capture. The active sites are mainly negative-charged N atoms and O atoms on functional groups, such as amine groups, azolate anions, phenolate anions, and imide anions [113,114]. Thus, it can be safely concluded that ILCOFs functionalized with these groups would obtain a high CO_2_ capacity, which is good for CO_2_ conversion. On the other side, with the high ionic conductivity and wide electrochemical window of ILs, the efficiency of the electrochemical conversion of CO_2_ could be improved through tuning the structure of ILs [115]. Special structures with photochemical properties could be decorated in ILCOFs to improve the efficiency of the photochemical conversion of CO_2_ [116,117]. Thus, more research is necessary on the development of ILCOFs and their applications for CO_2_ conversion. This review article gives academic researchers an overall understanding of ILCOFs and opens a door to develop novel ILCOFs materials for CCUS and the utilization of other gases.

## Data Availability

Not applicable.
